# Supplementary material for: The genome sequence of a dung beetle, Aphodius ( Calamosternus) granarius Linnaeus, 1767
Source: Wellcome Open Res. 2025 Sep 4;10:369. Originally published 2025 Jul 28. [Version 2] doi: 10.12688/wellcomeopenres.24306.2 (PMC12521904; doi:10.12688/wellcomeopenres.24306.2)
Supplement: Peer review history

# Reviewer report: Reviewer response for version 2

**DOI:** 10.21956/wellcomeopenres.27461.r132136

**Published:** 2025-10-14

**Authors:** Benjamin James Hunt

**Affiliations:** 1 University of Exeter, Exeter, UK

Mann and co-authors report the genome of the dung beetle *Aphodius granarius. *Assembly was conducted using a high quality and well-established pipeline as part of the Darwin Tree of Life Project, with the full process and associated metadata documented in the manuscript. The assembly achieves a good QV score and is very complete and low in duplication levels on the basis of its BUSCO score. Scaffolding to chromosome level appears to have been less successful than many of the ToL projects, with just under 70% of the genome assigned to chromosome-scale scaffolds. The genome is not yet annotated but this is underway - it would perhaps have been preferable to wait until this was completed before publication. 

 Two minor formatting notes:

 * In 'Background', *Aphodius granarius* is not italicised in the final sentence. 

 * In 'Data availability', the link to the ENA accession is broken.

Are sufficient details of methods and materials provided to allow replication by others?

Yes

Is the rationale for creating the dataset(s) clearly described?

Yes

Are the datasets clearly presented in a useable and accessible format?

Yes

Are the protocols appropriate and is the work technically sound?

Yes

Reviewer Expertise:

Entomology, bioinformatics

I confirm that I have read this submission and believe that I have an appropriate level of expertise to confirm that it is of an acceptable scientific standard.

# Reviewer report: Reviewer response for version 1

**DOI:** 10.21956/wellcomeopenres.26806.r130746

**Published:** 2025-09-02

**Authors:** Manuel Barrios-Izas

**Affiliations:** 1 University of San Carlos of Guatemala, Zacapa, Guatemala

Is the rationale for creating the dataset(s) clearly described?

 Yes, the authors describe this is a project that aim to sequence all named eukaryotic species in the Antlantic Archipielago of Britain and Ireland. The information will be useful for further research and is available on ENA.

 Are the protocols appropriate and is the work technically sound?

 Yes, the authors use standardized protocols from field through wet lab and bioinformatics lab. 

 Are sufficient details of methods and materials provided to allow replication by others?

- Figure 1, otherwise than db number, is a bad quality uninformative image.
- Please add the version of all the software used, as this will improve reproducibility.
- When you state that ‘amplicons were sequenced and compared to the BOLD database, confirming the species identification’, do you mean that you performed a BLAST search, or that you constructed a species tree?

Are the datasets clearly presented in a useable and accessible format?

 All datasets are available at ENA PRJEB61913

Are sufficient details of methods and materials provided to allow replication by others?

Partly

Is the rationale for creating the dataset(s) clearly described?

Yes

Are the datasets clearly presented in a useable and accessible format?

Yes

Are the protocols appropriate and is the work technically sound?

Yes

Reviewer Expertise:

Entomology, bioinformatics, molecular biology

I confirm that I have read this submission and believe that I have an appropriate level of expertise to confirm that it is of an acceptable scientific standard.
